# Beneficial effects of thermal waters on respiratory diseases: a systematic review

**DOI:** 10.1007/s00484-025-02865-z

**Published:** 2025-03-25

**Authors:** Mario Fontana, Matteo Vitali, Jole Del Prete, Salvatore Borzì, Angela Pozzoli, Katia Vitale, Andrea De Giorgi, Stefano Zanni, Serena Crucianelli, Carmela Protano

**Affiliations:** 1 Department of Biochemical Sciences, Sapienza University of Rome, Rome, 00185 Italy; 2 Department of Public Health and Infectious Diseases, Sapienza University of Rome, P.le Aldo Moro 5, Rome, 00185 Italy; 3 Department of Clinical Internal, Anesthesiological and Cardiovascular Sciences, Sapienza University of Rome, Rome, 00185 Italy

**Keywords:** Crenotherapy, Natural spring, Thermal water, Upper respiratory diseases, Lower respiratory diseases, Systematic review

## Abstract

Respiratory diseases are extremely common conditions worldwide with a high social and economic impact. The aim of this systematic review was to summarize the scientific evidence on the efficacy of thermal inhalation treatments to manage the signs and symptoms of all type of upper and lower respiratory diseases. The review was conducted according to the PRISMA recommendations. The protocol was registered in the PROSPERO platform (ID: CRD42024510869). The bibliographic search was performed using PubMed, Scopus and Web of Science databases without time limits up to January 2nd 2025. All experimental and semi-experimental studies conducted on humans, published in Italian and English, aimed to evaluate the effects of thermal inhalation treatments in the treatment of respiratory diseases were considered eligible. The quality of the studies was assessed using the CLEAR NPT scale. Overall, 27 studies were included, related to chronic and chronic-recurrent upper and lower respiratory tract diseases. The results agree on beneficial effects of thermal waters use, with an improvement in the sensation of nasal obstruction, rhinorrhea, muco-ciliary transport time and lung function parameters. The therapeutic effects determined by thermal inhalation treatments is attributed to the composition and biochemical activity of the different waters, which lead, among other effects, to a regularization of the activity of the immune system. The results, although agreeing and encouraging, cannot be definitive due to the limitations of the studies included, especially their low quality and heterogeneity. Therefore, further clinical studies should be conducted using more appropriate methodologies, study designs and statistical analysis techniques.

## Introduction

Thermal Waters (TW) are mineral waters that flow from a source or more bore holes or springs, originating from a geologically and physically protected underground. According to their temperature, they are classified into hypothermal (20–30 degrees), homeothermal (30–40 degrees), and hyperthermal (> 40 degrees). Although most TW are hypertonic solutions, their composition varies according to the concentration of minerals and salts contents. The variety in their composition is responsible for their different “bio properties”, that make them more or less indicated for the treatment of one or more diseases (Zajac 2021). For example, sulfur TW have been recognized to have anti-inflammatory properties and, consequently, they are suitable in the treatment of chronic diseases and recurrent infections (Prandelli et al. 2013). The anti-inflammatory action of TW has been known since ancient times. Indeed, the ancient Greeks already employed TW for the treatment of various inflammatory disorders (Bujs et al. 2019). In recent years, the scientific community has shown a strong interest in the use of TW as a therapeutic treatment for different pathologies. To date, several clinical trials have been performed for investigating the beneficial effects of TW on dermatological (Costantino and Filippelli 2014; Protano et al. 2024), osteoarticular (Fioravanti et al. 2011; Protano et al. 2023), gastroenterological (Quattrini et al. 2016), urological (Vitali et al. 2023) and respiratory pathologies, all with positive outcomes. Among the studied pathologies, the respiratory diseases are steadily increasing and are among the leading causes of morbidity and mortality worldwide. According to a 2017 estimate, overall, 544.9 million people were affected by a chronic respiratory disease (GBD Chronic Respiratory Disease Collaborators 2020). In particular, in recent decades, the prevalence of asthma and other atopic diseases, such as allergic rhinitis, has increased (Zhang et al. 2023). As a matter of fact, about 20% of the world’s population is affected by these conditions (Dierick et al. 2020). Asthma and COPD are among the most common chronic respiratory diseases. These two pathologies represent themselves a great burden for healthcare system both in term of cost (Dierick et al. 2020) and from a social perspective. For example, COPD flare-ups have a negative impact on the well-being and quality of life of affected individuals (MacLeod et al. 2021). In addition, although to a lesser extent, recurrent upper respiratory tract infections, such as allergic and nonallergic rhinitis, acute and chronic rhinosinusitis with or without nasal polyps, have a significant socio-economic impact (Bhattacharyya 2003). All of these diseases are ubiquitous both considering age and geographical distribution (Bousquet et al. 2009).

The high prevalence of respiratory diseases and their strong socio-economic impact led to the search of additional therapies, such as crenotherapy and SPA therapy (swimming training in a hot-spring, inhalation of iodine salt solution and mud therapy). The latter has shown to have numerous beneficial effects on asthma (Ashida et al. 2002; Tanizaki et al. 1992, 1993a, b; Mitsunobu et al. 1992, 1997; Vu and Mitsunobu 2004) and steroid-dependent intractable asthma (Tanizaki et al. 1993a, b; Hosaki et al. 1996), reducing the pharmacological costs necessary for managing this condition (Ashida et al. 2005). In COPD, the use of spa therapy has also demonstrated improved disease control (Takata et al. 2008). TW treatment is also a key adjunctive therapy for respiratory problems (Zajac 2021). According to the clinical trials conducted, TW action has been hypothesized to be related to the body’s immune response through anti-inflammatory and mucolytic action (Nimmagadda et al. 2022). Besides, analgesic, antioxidant and antibacterial effects have been underscored (Viegas et al. 2019). In addition to the recognized beneficial effects, TW-based treatments are non-invasive, they have minimal adverse effects and no drug interactions. Although numerous studies have already investigated the use of crenotherapy as a treatment for respiratory diseases, a critical evaluation of the evidence in a systematic review is needed. Indeed, systematic reviews of scientific literature on this issue have been performed, but they were limited to investigate the use of TW for the treatment of upper respiratory tract diseases (Keller et al. 2014) or just for lower respiratory tract diseases such as COPD and asthma (Calzetta et al. 2024) or asthma (Fesyun et al. 2023). To our knowledge, no recent systematic review summarizing the scientific evidences on the use of TW inhalation treatments on all known respiratory diseases has been performed yet. The present study aims to evaluate and summarize the scientific evidence on the efficacy of TW inhalation treatments to manage signs and symptoms of respiratory diseases.

## Materials and methods

### Research strategy

The present systematic review was realized according to the PRISMA (Preferred Reporting Items for Systematic Reviews and Meta-Analyses) Statement (Page et al. 2021). The protocol was recorded on the PROSPERO platform with the following ID: CRD42024510869.

The following bibliographic and citation databases were consulted: PubMed, Scopus and Web of Science (Science and Social Science Citation Index). We used the keywords with Boolean operators as AND–OR utilizing the following query: (“thermal water*” OR “thermal treatment*” OR “add-on therapy”OR “inhalation* of thermal water*” OR “inhalatory treatment* of thermal water” OR “thermal aerosol” OR “nebulized thermal water*”OR “thermal source*” OR “ thermal spring*” OR “sulphurous thermal water” OR “sodium-chloride-sulfide thermal water*” OR “iodine-bromide thermal water*” OR “bicarbonate-magnesium-calcium thermal water*” OR “thermal water with sodium-chloride” OR “fluorohydrous-sulphurous-hydrofluoric radioactive thermal water*” OR “thermal water* with sulphur” OR “hyperosmolar thermal water*” OR “hyposmolar thermal water*”) AND (“respiratory disease*” OR “airway disease*” OR “respiratory disorder*” OR “airway disorder*” OR “acute respiratory disease*” OR “chronic respiratory disease*” OR “chronic bronchitis” OR “chronic obstructive pulmonary disease” OR “disease* of the upper airway” OR “disease of the lower airway” OR “nasal obstruction” OR “acute airway inflammation” OR “chronic airway inflammation” OR “allergic rhinitis” OR “asthma” OR “rhinosinusitis”).

The research contained all the articles published up to January 2nd 2025.

### Inclusion and exclusion criteria

In the present review, we considered all the papers published in Italian and English language aiming assessing the effects of TW based treatments on human respiratory diseases. We included any experimental and quasi-experimental study on humans, whereas case reports, case series, letters to editors, commentaries, editorials, reviews and observational studies were excluded. Grey literature was not considered. References of further critical and systematic reviews and/or meta-analyses have been evaluated in order to retrieve other published literature. We excluded any article not matching the inclusion criteria.

PICOS model was employed for structuring the research question, as follows:

- Population: all genders, all ages, with acute and chronic respiratory diseases.
- Intervention: use of TW in patients with respiratory diseases.
- Control: age-, gender- and condition-matched control group.
- Outcomes: understand if use of TW has beneficial effects on symptoms and signs in patients affected by respiratory diseases.
- Study: quasi-experimental and experimental studies on humans. All studies that did not satisfy the inclusion criteria were excluded.

We transferred all the references of the selected articles on Zotero citation management software (RRID: SCR_013784) to remove any duplicates and to assess the relevance of each article.

Four researchers (S.B., J.D.P., A.P. and K.V.) independently checked the potentially eligible studies by reading titles and abstracts. Two content experts (M.V. and C.P.) assisted with the screening and reviewing process and three graduate students (S.C., A.D.G. and S.Z.) assisted with the searching, screening, evaluating process. Afterwards, the four investigators evaluated independently the full text of each article included. The group debated and solved any dissent about the selected papers or asked the subject matter expert of the team (M.F.) to resolve the conflict.

### Risk of bias assessment

Four investigators (S.B., J.D.P., A.P., K.V.) independently estimated the quality of any single study through the CLEAR NPT checklist (Checklist to Evaluate a Report of a Non pharmacological Trial). This checklist is based on the Delphi method and it is composed of 10 questions with three answer options each (Yes, No, Not Reported). These questions allowed to determine the risk of bias for each study (High, Medium and Low Bias Risk). Particularly, each affirmative answer corresponds to one point. From 10 to 8 points means a low bias risk, from 7 to 5 stands for a medium bias risk and lower than 5 a high bias risk. The group of four researchers debated and solved any dissension about the score obtained for each study. The evaluation of quality was reported into the data extraction Tables 1 and 2. Furthermore, information on author, year of publication, country, study design, TW characteristics, sample size and characteristics of study population, respiratory diseases, treatments, outcomes and results have been reported for each article in Tables 1 and 2.

**Table 1:** – characteristics of the studies included in the systematic review reporting data on upper respiratory diseases

Author Year Country Study Design Thermal Water Characteristics	Sample Size Study Population Tot; *n* interventions and *n* controls; mean age ± SD years, *n* Females (%) and *n* Males (%)	Upper Respiratory Diseases	Outcomes	Interventions and controls	Results	Quality of included studies (clear NPT score) High/Medium/Low Bias Risk
Varricchio et al. 2013 Italy Randomized single blind clinical trial Salso-sulphide themal water of Agnano	107; 56 interventions and 51 controls; 4.5 ± 1.2 years; 37 Females (34.6%) and 70 Males (65.4%)	Recurrent respiratory infections (RRI)	Evaluation of the effects of salso-sulphide thermal water nasal irrigation on recurrent respiratory infections prevention and on improvement of signs and symptoms in children	Interventions: inhalation of thermal water with nasal washing by Rinojet (ASEMA srl, Milan, Italy) b.i.d. for 12 days; nasal washing lasted 2 min per nostril and, immediately before it, children inhaled 1 L of water by stream inhalation for 10 min Controls: inhalation of isotonic saline water with nasal washing by Rinojet (ASEMA srl, Milan, Italy) b.i.d. for 12 days; nasal washing lasted 2 min per nostril and, immediately before it, children inhaled 1 L of water by stream inhalation for 10 min	Significant reduction of the number of respiratory infections (from 8.2 ± I.2 to 3.1 ± 1.6, *p* < 0.001), nasal symptoms (from 2.7 ± 0.7 to 0.6 ± 0.7, *p* < 0.001), turbinate hypertrophy (from 1 ± 0.9 to 0.6 ± 0.9) and adenoidal hypertrophy (from 73.2–46.4%), blockage of ostiomeatal complex (from 26.5–8.9%, *p* < 0.001); no significant reduction of neutrophil and bacterial count	Medium Bias Risk
Marullo e Abramo 1999 Italy Controlled double-blind clinical trial Sulphurous-arsenical-ferruginous thermal water of Terme di Levico	55; 37 interventions and 14 controls; 39.5 years; 30 Females (54.5%) and 25 Males (45.5%)	Aspecific phlogosis of the upper respiratory tract (URT)	Evaluation of the effects of thermal water inhalation on the functional capacity of the nasal mucosa, monitoring nasal respiratory function, mucociliary clearance, cytological study of the nasal smear, biochemical study of nasal mucus, tasting the potential immunological respiratory mucosa defense capacity	Interventions: 12-day inhalation cycle of thermal water, with daily steam inhalations in a common environment for 10 min and nasal aerosols for 10 min (average micelle size: 7 micron) Controls: 12-day inhalation therapy cycle of drinking water, with daily steam inhalations in a common environment for 10 min and nasal aerosols for 10 min	Statistically significant improvement in nasal flow on active anterior rhinomanometry and significant reduction in nasal resistance after inhalation therapy in interventions (*p* < 0.01), while non-significant changes in controls; significant reduction in mucociliary transport times from an average of 19.2 min to 11.9 min in interventions (*p* < 0.01); non-significant reduction in controls; significant reductions in neutrophilic leukocytes and bacterial counts in interventions (*p* < 0.01); no significant changes in eosinophil lymphocytes, macrophages and monocytes nor in the phenomena of squamous metaplasia in both groups; in the immunochemical analysis of nasal mucus, variation in the electrolyte composition of the nasal secretion in interventions and not in controls; significant increase in the concentration of albumin and non-secretory Ig in interventions (*p* < 0.02); marked increase in concentration of the secretory piece (from 195.22 mg/dl to 285.16 mg/dl, highly significant difference *p* < 0.001)	Low Bias Risk
Marullo and Abramo 2000 Italy Controlled double-blind clinical trial Radioactive-fluoridated oligominerals waters from the Merano Spa	46; 40 interventions and 6 controls; 59.3 years; 21 Females (45.7%) and 25 Males (45.3%)	Aspecific upper respiratory tract chronic catarrhal phlogosis	Evaluation of the effects of thermal water inhalation by monitoring nasal ventilation function mucociliary clerance, nasal mucus cytological study, tasting the potential immunological respiratory mucosa defense capacity	Interventions: 12-day inhalation cycle of thermal water, with daily steam inhalations in a common environment for 10 min and nasal aerosols for 10 min (average micelle size: 7 micron) Controls: 12-day inhalation therapy cycle of drinking water, with daily steam inhalations in a common environment for 10 min and nasal aerosols for 10 min	Statistically significant improvement in nasal airflow and nasal resistance in interventions (*p* < 0.01), but not significant in controls; significant reduction in mucociliary transport times from an average of 18.1 min to 11.7 min (*p* < 0.01) in interventions, not significant in controls; significant reduction in neutrophils and bacterial carpet in interventions (*p* < 0.05), but no significant changes in eosinophilic lymphocytes, macrophages and monocytes nor in the phenomena of squamous metaplasia in both groups; variation in electrolyte composition of the nasal secretion in the interventions, with a significant increase in the concentration of albumin and non-secretory Ig (*p* < 0.02); marked increase in concentration of the secretory piece (from 171.55 mg/dl to 199.07 mg/dl, highly significant difference *p* < 0.02)	Low Bias Risk
Vassallo et al. 2009 Italy Quasi-Experimental trial sulphurous-bicarbinate-calcium-magnesium thermal water of thermal station “Telese terme”	40; 38.6 years; 12 Females (30%) and 22 Males (70%)	Catarrhal pathologies and inflammatory diseases of the upper aero-digestive tract	Evaluation of clinical subjective symptoms (dry mouth, burning pharynx, odynophagia, cough) and laboratory (cytological examination and mucociliary transport test) efficacy of a treatment with thermal water	A cycle of direct steam jet inhalations and nasal aerosols once a day for 12 days	Statistically significant reduction in subjective symptoms (dry mouth, burning pharynx, odynophagia, cough; *p* < 0.01); significant reduction in hyperemia of the pharyngeal and nasal mucosa, respectively (*p* < 0.05, *p* < 0.01); significant reduction of anterior rhinorrhea (*p* < 0.05), posterior rhinorrhea (*p* < 0.01) and pharyngeal discharge (*p* < 0.001); significant positive changes in hypertrophy of the inferior turbinates (*p* < 0.01)	Medium Bias Risk
Magrone et al. 2016 Italy Quasi-Experimental trial Salt-bromide-iodine thermal water of thermal station “Margherita di Savoia” Sponsorized	102; 58 (24–86) years; 55 Females (53.9%) and 47 Males (46.1%)	Chronic upper respiratory tract infections (cURTI)	Comparison of peripheral blood serum cytokine values (Th1-related cytokines such as IL-12, IL-2 and IFN-γ, Th-2-related cytokines such as IL-4, the IL-10/IL-17/IL-21 axis, the pro-inflammatory cytokines IL-1β, TNF-α, IL-6, IL-8) and clinical evaluation (mainly frequency of infectious episodes)	Inteventions: single cycle of inhalatory therapy with nebulised thermal water (TW) under form of microparticles, prewarmed at 37 °C, once a day for 10 min, for 2 weeks Controls: no treatment	Reduction in infectious episodes frequency in both groups (no infectious episodes at T2 in comparison to 3 events at T0 in 33 elderly cURTI patients). In 15 of them, only one episode was recorded at T2 when compared to 2.2 infectious events at T0, no episodes at T2 versus 4 events at T0 in 29 young cURTI patients, in the remaining 19 cases, 1.6 episodes at T2 vs. 4 events at T0); increase of IL-12 at T2 (*p* < 0.01 in both groups), of IL-17 at T2 (*p* < 0.05 in elderly patients and *p* < 0.01 in young patients), of IL-21 at T2 (*p* < 0.01 in elderly patients and *p* < 0.05 in young patients), of IL-1β at T2 (*p* < 0.01 in elderly patients and *p* < 0.05 in young patients), of TNF-α at T2 (*p* < 0.001 in elderly patients and *p* < 0.05 in young patients), of IL-6 (*p* < 0.001 in both groups) and of IL-8 (*p* < 0.01 in elderly patients and *p* < 0.001 in young patients); decrease of IL-2 at T2 (*p* < 0.05 in elderly patients and *p* < 0.01 in young patients), of IFN-γ at T2 (*p* < 0.001 in both groups), of IL-4 at T2 (*p* < 0.001 in elderly patients and *p* < 0.05 in young patients) and of IL-10 at T2	Medium Bias Risk
Cristalli et al. 1996 Italy Randomized clinical trial Sulphur-sulphate-alkaline-earth metals water (Terme dei Papi Viterbo)	50; 40 interventions and 10 controls; 26–69 years	Chronic inflammation of upper respiratory airways	Evaluation of the prophylactic-therapeutic effect of crenotherapy by analyzing rhinocytograms, using plasma cells, neutrophils and bacterial flora as comparison parameters	Interventions: inhalation of thermal water once a day for 12 days Controls: inhalation of physiological water once a day for 12 days	Plasma cells increase in interventions (65%) and in controls (40%); decrease in neutrophils in interventions (60%) and in controls (50%); bacterial flora decrease in interventions (65%, *p* = 0.05) and in controls (30%); total disappearance of squamous metaplasia only in interventions (30%)	High Bias Risk
Pollastrini et al. 1996 Italy Controlled clinical trial Sulphurus-sulphate-bicarbonate-carbonic-alkaline-earth metals water of Bullicame spring	50; 40 interventions and 10 controls; 48.42 years; 27 Females (54%) and 23 Males (46%)	Chronic nonallergic catarrhal rhinopharyngopathy	Evaluation of the effects of the sulphurus-sulphate-bicarbonate-carbonic-alkaline-earth metals water on mucociliary transport (tTMC) and nasal perviousness alterations	Interventions: inhalation using a direct jet of thermal water at 37° C for 10 min with a break of at least 5 min between the two sessions, for 12 days, with two daily sessions Controls: inhalation of aerosol using special nasal hairpins for 10 min with a break of at least 5 min between the two sessions, for 12 days, with two daily sessions	Improvement in tTMC in 29 interventions and 2 controls; no significant differences in tTMC evidenced in 9 interventions and 8 controls; declination in tTMC evidenced in 2 interventions; improvement of nasal global perviousness resistance in 21 (53.8%) interventions and 1 (10%) control; no significant difference of nasal global perviousness resistance in 13 (33.3%) interventions and 6 (60%) controls; decline of nasal global perviousness resistance in 5 (12%) interventions and 3 (30%) controls	Medium Bias Risk
Staffieri and Abramo 2007 Italy Quasi-Experimental trial Sulphurous-arsenical-ferruginous thermal water of Levico Spa	37; 40 years; 20 Females (54.1%) and 17 Males (45.9%)	Chronic sinonasal disease	Evaluation of the effects of sulphurous-arsenical-ferruginous thermal water inhalations on nasal respiratory flow, mucociliary transport, nasal cytology and chemo-physics of nasal mucus	Interventions: 12-day course of sulphurous-arsenical-ferruginous thermal water warm vapour inhalations (38 °C to 20 cm from patient’s face for 10 min), followed by nasal aerosol of the same thermal water (7 microns micelle, for 10 min) Controls: no treatment	Significant improvement in nasal flow (from 558.4 ml/s ± 98.9 ml/s to 705.6 ml/s ± 104.7 ml/s, *p* < 0.01); significant reduction of nasal resistance (from 0.47 Pa ± 0.21 Pa to 0.28 Pa ± 0.091 Pa, *p* < 0.01), in the mean mucociliary transport time (from 19.3 min ± 8.20 min to 11.9 min ± 6.45 min, *p* < 0.01), in the concentration of neutrophils in 44% of the cases (*p* < 0.001), of nasal bacterial presence in 56.7% of the cases (*p* < 0.001); significant increase (*p* < 0.05) of plasma cells concentrations in 50% of the considered cases	High Bias Risk
Passariello et al. 2012 Italy Quasi-Experimental trial Sulfate-sodium-chloride thermal water (Therapy was performed in a thermal site in the island of Ischia)	60; 3.4 ± 1.0 years; 32 Females (53.3%) and 28 Males (46.7%)	Chronic rhinosinusitis (CRS)	Evaluation of the effects of crenotherapy with sulfate-sodium-chloride water on clinical symptoms (SN5 and QoL score) and on mucosal markers of inflammation (TNF alpha, calprotectin, hBD-2) in children with chronic rhinosinusitis	Interventions: 15-day of sulfate-sodium-chloride thermal water inhalations by nasal aerosol (15 min/day) Controls: no treatment	Significant improvement in quality of life (from 4.2 ± 1.1 to 6.6 ± 1.0, *p* < 0.001) with a significant reduction (*p* < 0.05) of subjects presenting symptoms of nasal obstruction (100% versus 40%,), nasal discharge (33% versus 13%), facial pain (30% versus 10%) and smell (60% versus 20%); significant reduction in concentrations of TNF alpha (from 0.14 ± 0.02 to 0.08 ± 0.01, *p* < 0.001), calprotectin (from 2.9 ± 1.0 to 1.9 ± 0.5, *p* < 0.001) and hBD-2 (from 2.0 ± 0.1 to 0.9 ± 0.6, *p* < 0.001)	High Bias Risk
Salami et al. 2010 Italy Randomized double-blind clinical trial Sulphurous thermal water of Tabiano SPA	80; 40 interventions and 40 controls; 26–58 years; 35 Females (43.7%) and 45 Males (56.3%)	Chronic rhinosinusitis (CRS)	Evaluation of the effects of sulphurous thermal water on clinical symptoms (VAS score), nasal mucociliary transport time and cytology (IgA and IgE) in patients with chronic rhinosinusitis	Interventions: inhalation of thermal water (38 °C, at a distance of 20 cm from the patient’s face for 10 min) for 12 days Controls: inhalation of physiological solution (38 °C, at a distance of 20 cm from the patient’s face for 10 min) with nasal irrigations for 12 days	Significant improvement in symptomatology (VAS score) (at 12 days: 1.7 ± 0.18 versus 6.9 ± 0.51, at 3 months: 1.8 ± 0.22 versus 7.1 ± 0.59, *p* < 0.05); significant reduction of serum concentration of IgE (at 12 days: 76.27 ± 26.3 mg/dl versus 97.44 ± 45.4, at 3 months: 75.48 ± 26.1 mg/dl versus 98.37 ± 41.4, *p* < 0.05) and total nasal resistances; no changes in nasal mucociliary transport time (at 12 days: 11.54 ± 1.59 min versus 17.38 ± 1.83 min, at 3 months: 11.46 ± 2.07 min versus 17.43 ± 2.01 min) and in IgA serum titers (at 12 days: 231.09 ± 120.3 mg/dl versus 220.44 ± 114.4 mg/dl, at 3 months: 235.44 ± 118.5 mg/dl versus 214.51 ± 111.8 mg/dl)	Low Bias Risk
Passali et al. 2008a Italy Randomized clinical trial Salso-bromo-iodic isotonic thermal water (Acquasal spray) of Salsomaggiore Terme	55; 30 interventions and 25 controls; 40.5 ± 12.5 years; 27 Females (49%) and 28 Males (51%)	Chronic rhinosinusitis or nasal polyposis	Evaluation of the clinical (Nasal Symptomatology Score), instrumental (rhinomanometry) and laboratory (mucociliary transport times and citology) efficacy of a treatment with thermal water as nasal spray versus saline on chronic rhinosinusitis with/without nasal polyps	Interventions: thermal water nasal spray for 4 times/day for 4 weeks Controls: nasal spray of saline physiological solution for 4 times/day for 4 weeks	Improvement of nasal obstruction in interventions (*p* = 0.080); significant improvement in interventions in headhache (*p* < 0.001, highly significant level), rhinorrhea (*p* = 0.006), hiposmia (*p* = 0.018), pain (*p* = 0.016), nasal mucosa appearance (71.4% in interventions and 28.6% in controls, *p* = 0.007) and crusts (70.4% in interventions and 27.3% in controls, *p* = 0.007), rhinomanometric values (*p* = 0.011), mucociliary transport times (*p* = 0.006); no significant difference in nasal citology between interventions and controls; significant improvement of percentage of epithelial cells (78.3% in interventions and 33.3% in controls, *p* = 0.015); significant decrease of globlet cells in interventions (*p* = 0.002)	Medium Bias Risk
Passali et al. 2008b Italy Randomized clinical trial Salt-bromine-iodic thermal water of Salsomaggiore Terme	120; 60 interventions and 60 controls; 16–65 years; 52 Females (43%) and 68 Males (57%)	Recurrent or chronic rhinosinusitis with/without I degree nasal polyposis according to the Lund-Mckay classification	Evaluation of the clinical, instrumental (rhinomanometry, acoustic rhinometry) and laboratory (mucociliary transport times and citology) efficacy of thermal water versus nasal douching cycle and aerosol with saline in the recurrent and chronic nasosinusal pathologies treatment	Interventions: daily sessions of thermal steam inhalations at 37° for 10 min, thermal aerosol for 10 min, nasal douching for 5 min per nostril for 14 days Controls: nasal douching cycle and aerosol with saline twice a day for 14 days at the Rhinologic Centre of the ENT Clinic of Siena University	Significant improvement in interventions of nasal obstruction (*p* < 0.001), rhinorrea (*p* < 0.001), number of nocturnal arousals (*p* < 0.001), nasal mucosa congestion (*p* = 0.005), nasal secretion (*p* = 0.005) and mucociliary transport time (*p* < 0.001); no significant differences for the symptoms hyposmia, headache and sneezing; normalization of cytological picture in 26 patients of 31 in interventions, and in 8 patients of 31 in controls; decrease of neutrophils and eosinophils at the rhinocytogram in interventions (from 19 to 6 patients, *p* < 0.001) and in controls (from 16 to 12 patients), significant decrease of goblet cells in interventions (*p* < 0.001); significant decrease of nasal resistance in interventions (*p <* 0.001) and in controls (*p* = 0.002); significant improvement of rhinomanometry and acoustic rhinometry (*p* < 0.001)	Medium Bias Risk
Ottaviano et al. 2011 Italy Randomized double-blind clinical trial Sulfurous, salty, bromic, iodic (SSBI) thermal water of Sirmione Spa Sponsorized	80; 40 interventions and 40 controls; 18–65 years	Nonallergic chronic rhinosinusitis	Comparison of nasal resistance, endoscopic and cytology effects of nasal irrigations with thermal water or isotonic sodium chloride solution in patients with nonallergic chronic rhinosinusitis	Interventions: nasal irrigations with SSBI thermal water for 1 month Controls: nasal irrigations with isotonic sodium chloride solution (ISCS) for 1 month	Significant endoscopically assessed clinical improvement in interventions (*p* = 0.005) and in controls (*p* = 0.01); no sign of Staphylococcus aureus in interventions (from 5 patients to 0) and in controls (from 4 patients to 0); significant total nasal resistance decrease in interventions (from 0.22 ± 0.20 Pa, *p* = 0.30 to 0.15 ± 0.08 Pa, *p* = 0.01) and no significant change in controls (from 0.17 ± 0.24 Pa, *p* = 0.30, to 0.14 ± 0.06 Pa, *p* = 0.25); no significant change in the HSS + rate (Hyperchromatic Supranuclear Stria, a valid marker of the anatomical and functional integrity of nasal mucosal hair cells) in interventions (from 27% ± 13%, *p* = 0.30 to 25% ± 17%, *p* = 0.84) and in controls (from 22% ± 16%, *p* = 0.30 to 26% ± 13%; *p* = 0.26)	Low Bias Risk
Ottaviano et al. 2012 Italy Randomized double-blind clinical trial Sulfurous-arsenical-ferruginous thermal water of Levico Spa	70; 35 interventions and 35 controls; 18–65 years; 48 Females (68.6%) and 22 Males (31.4%)	Nonallergic chronic rhinitis	Evaluation of the endoscopic, nasal resistance and cytology effects of nasal irrigations with thermal water or isotonic sodium chloride solution in smokers with nonallergic chronic rhinosinusitis	Interventions: nasal irrigations with sulfurous-arsenical-ferruginous thermal water, 20 mL/day for 1 month Controls: nasal irrigations with isotonic sodium chloride solution, 20 mL/day for 1 month	Statistical trend toward lower nasal resistances in interventions (from 0.10 ± 0.38 to 0.08 ± 0.03 after first follow-up, at the end of 1 month of treatment, *p* = 0.07 and to 0.08 ± 0.02 at the second follow-up, two months after completing the treatment, *p* = 0.052) and no statistically significant difference in controls; significant increase of ciliated cells in interventions only at first follow-up (from 9.31 ± 8.99 to 11.54 ± 7.33, *p* = 0.003); significant increase of neutrophil count in controls at second follow-up (from 8.85 ± 12.89 to 9.11 ± 6.31, *p* = 0.0001) improvement of olfactory threshold in controls at first follow-up (from 7.77 ± 1.49 to 8.32 ± 0.70, *p* = 0.04); significant decrease of nasal resistance in interventions rather than in controls at both follow-up (*p* = 0.001 and *p* = 0.0003, respectively); significant increase of ciliated cells and neutrophils in interventions rather than in control after first follow-up (*p* = 0.059 and *p* = 0.02, respectively); significantly higher olfactory threshold in controls than in interventions at both follow-up (*p* = 0.007 and *p* = 0.01, respectively); significant improvement in clinical picture at nasal endoscopy in interventions (*p* = 0.03)	Low Bias Risk
Passali et al. 2013 Italy Quasi-Experimental trial Radioactive hydrofluoric oligomineral thermal water of Merano Therme	33; > 12 years; 10 Females (30%) and 23 Males (70%)	Chronic rhinosinusitis Persistent allergic rhinitis Vasomotor rhinitis	Evaluation of the improvement of mucociliary function and nasal cytology with nasal inhalatory treatment with thermal water and of the efficacy of SPA therapy with radioactive water as alternative choice in chronic inflammatory diseases of the upper airways, nonresponsive to pharmacological therapy	Interventions: nasal irrigations with SSBI thermal water for 1 month Controls: nasal irrigations with isotonic sodium chloride solution (ISCS) for 1 month	No significant changes for nasal resistance values; significant improvement of mucociliary function (*p* = 0.039) and in nasal cytology (from 14 patients with inflammatory cells infiltrate, with or without bacterial presence, to 5)	High Bias Risk
Pagani et al. 2011 Italy Quasi-Experimental trial CO2-enriched thermal water of Fonti di Rabbi Spa Centre	50; 25 interventions and 25 controls; 45.95 years; 25 Females (55%) and 25 Males (55%)	Allergic rhinitis	Evaluation of the clinical (Symptom scale) and laboratory (quantitative sandwich enzyme immunoassay technique and cytometric analysis) efficacy of a treatment with thermal water	Interventions: inhalations of thermal water once a day, 6 days a week for 2 consecutive weeks + inhalations of saline solution Controls: inhalations of thermal water once a day, 6 days a week for 2 consecutive weeks + inhalations of saline solution	Statistically significant difference of symptoms between patients and healthy controls before and after each treatment; significant reduction of IL-3, IL-5 and eotaxin levels in the nasal lavage fluid of patients after thermal water treatment, compared with those before and after saline treatment, respectively (IL-3: 38.7 ± 1.3 vs. 55.0 ± 2.2 IU/mL, *p* < 0.001; IL-3: 38.7 ± 1.3 vs. 52.4 ± 1.6 IU/mL, *p* < 0.01; IL-5: 24.9 ± 0.8 vs. 36.2 ± 1.0 IU/mL, *p* < 0.001; IL-5: 24.9 ± 0.8 vs. 33.8 ± 1.8 IU/mL, *p* < 0.01; eotaxin: 296.0 ± 11.3 vs. 477.0 ± 19.6 0 IU/mL, *p* < 0.001; eotaxin: 296.0 ± 11.3 vs. 445.0 ± 16.4 IU/mL, *p* < 0.01). No differences were observed in healthy controls; statistically significant difference of percentage of eosinophil, in patients and controls, respec- tively, before treatment (50 ± 13 vs. 18 ± 10, *p <* 0.001), after saline treatment (48 ± 9 vs. 19 ± 7, *p* < 0.001), and after CO2-enriched water (30 ± 8 vs. 15 ± 6, *p* < 0.001)	Medium Bias Risk
Ciprandi et al. 2016 Italy Quasi-Experimental trial Sulphurous and salso-bromo-iodic thermal waters of Comano Terme	30; 40.9 years; 17 Females (57%) and 13 Males (43%)	Allergic Rhinitis	Evaluation of the effects of Comano thermal water inhalation on TSS (Total Symptom Score) and on VAS (Visual Analogue Scale)	Interventions: inhalation of 1 L of solution, supplied by Asema inhaler, for 10 min Controls: no treatment	Significant decrease (*p* < 0.001) of TSS (Total Symptom Score) values (from M 6, IQR 4.5–8 to M 3, IQR 1–5); significant increase (*p* < 0.001) of VAS (Visual Analogue Scale) values (from M 2, IQR 2–2 to M 8, IQR 6.8–9), but significant decrease (*p* < 0.001) at follow-up (M 4, IQR 2.8–5)	Medium Bias Risk
Passali et al. 2016 Italy Randomized clinical trial Radioactive oligomineral water of the Merano hot spring	90; 54 interventions and 36 controls; 14–80 years	Perennial allergic rhinitis	Assessment of the clinical (SNOT score) and laboratory (cytology, nasal resistance, mucociliary transport time) efficacy of nasal radioactive oligomineral water vapours inhalations from the Merano hot spring versus mometasone furoate nasal spray	Interventions: inhalation of radioactive thermal oligomineral waters for 14 days Controls: topic treatment with mometasone furoate nasal spray for 14 days	No significant changes in clinic (SNOT score) and cytology between interventions and controls; significant increase (*p* = 0.049) of nasal air flow in interventions (from 482 cc/s to 528 cc/s) and in controls (from 470 cc/s to 492 cc/s); decrease of nasal resistance in interventions (from a mean of 0.25 Pa/cc to 0.23 Pa/cc, *p* = 0.76) and in controls (from 0.34 Pa/cc to 0.26 Pa/cc, *p* = 0.093); significant improvement in mucociliary transport time in interventions (from a mean of 13 min to a mean of 12 min, *p* < 0.001) and in controls (from a mean of 14 min to a mean of 13 min, *p* = 0.003); increase in ciliated cells in interventions (from a mean of 30 to 33.47 per microscope field) and in controls (from a mean of 25.7 to 30 per field); decrease in interventions of neutrophils (from a mean of 8 to 4 per field) and eosinophils (from 0.26 to 0.065 per field); decrease in controls of muciparous goblet cells (from 30 to 27.4 per field)	Medium Bias Risk
Barbieri et al. 2002 Italy Randomized clinical trial Iodine Bromide Water of Salsomaggiore	80; 40 interventions and 40 controls; 23–76 years; 44 Females (55%); 36 Males (45%)	Positivity to Dermatophagoides Pteronyssinus and/or Dematophagoides Farinae at allergic studies	Evaluation of the effects of iodine bromide water of the thermal baths of Salsomaggiore on symptoms, IgA and IgE values and Mucociliary Transport Test	Interventions: endonasal Acqua Sal spray, 7 times/day for 30 days Controls: endonasal oily dops, 7 times/day for 30 days	Symptomatic improvement in 80 (100%) participants (from the mean of 7.5 to 3 in the symptomatic scale); reduction in total IgE in interventions (from 141 ± 116.6 to 103 ± 88.2) and increase in controls (from 135.8 ± 102.5 to 139.6 ± 103.6); increase in total IgA (mg/dl) in interventions (from 255.9 ± 49.2 to 263.5 ± 49.1) and decrease in controls (from 214.6 ± 55.9 to 209.3 ± 56.6); reduction of the values in the Mucociliary Transport Test in interventions (from 19500 ± 3494 to 13675 ± 1457) and in controls (from 19150 ± 3231 to 17875 ± 2972)	Medium Bias Risk
Miraglia Del Giudice et al.^*^ 2011 Italy Randomized clinical trial Hyper-mineral chloride sodium water of “Lacco Ameno”	34; 18 interventions and 16 controls; 9.6 ± 1.8 years; 20 Females (58.8%) and 14 Males (41.2%)	Allergic rhinitis and intermittent asthma during the period of natural exposure to Parietaria pollen	Evaluation of the effects of thermal water nasal irrigation on nasal symptoms TSS (Total Symptom Score) and airway inflammation (using spirometry and exhaled nitric oxide, FeNO)	Interventions: inhalation of thermal water aerosol by nasal adapter, daily, for 15 days per month, for 3 consecutive months Controls: micronized nasal douche with 0.9% NaCI (isotonic) solution, daily, for 15 days per month, for 3 consecutive months In addition, all children were treated on demand with cetirizine (0.5 gtt./kg/day once daily)	Rhinitis symptoms reduction, assessed by the TSS (Total Symptom Score), only in interventions; significant FeNO values decrease (*p <* 0.001) in interventions (19.2 ± 8.4) and in controls (53.4 ± 21.6); no differences in FEV-1 values in both groups	Medium Bias Risk

^*^ Miraglia Del Giudice et al. 2011 reported data on both upper and lower respiratory diseases and, thus, the article was included in both Tables 1 and 2

**Table 2:** – characteristics of the studies included in the systematic review reporting data on lower respiratory diseases

Author Year Country Study Design Thermal Water Characteristics	Sample Size Study Population Tot; *n* interventions and *n* controls; mean age ± SD years, *n* Females (%) and *n* Males (%)	Lower Respiratory Disease	Outcomes	Interventions and controls	Results	Quality of included studies (clear NPT score) High/Medium/Low Bias Risk
Miraglia Del Giudice et al.^*^ 2011 Italy Randomized clinical trial Hyper-mineral chloride sodium water of “Lacco Ameno”	34; 18 interventions and 16 controls; 9.6 ± 1.8 years; 20 Females (58.8%) and 14 Males (41.2%)	Allergic rhinitis and intermittent asthma during the period of natural exposure to Parietaria pollen	Evaluation of the effects of thermal water nasal irrigation on nasal symptoms TSS (Total Symptom Score) and airway inflammation (using spirometry and exhaled nitric oxide, FeNO)	Interventions: inhalation of thermal water aerosol by nasal adapter, daily, for 15 days per month, for 3 consecutive months Controls: micronized nasal douche with 0.9% NaCI (isotonic) solution, daily, for 15 days per month, for 3 consecutive months In addition, all children were treated on demand with cetirizine (0.5 gtt./kg/day once daily)	Rhinitis symptoms reduction, assessed by the TSS (Total Symptom Score), only in interventions; significant FeNO values decrease (*p <* 0.001) in interventions (19.2 ± 8.4) and in controls (53.4 ± 21.6); no differences in FEV-1 values in both groups	Medium Bias Risk
Sanguinetti et al. 1990 Italy Quasi-Experimental trial Sulphureous thermal water of Rofanello	10; 13–39 years; 4 Females (40%) and 8 Males (60%)	Bronchial Asthma	Evaluation of the effects of thermal water inhalation on changes in the caliber of the airways	Interventions: inhalation for 5 min of an aerosol of hypotonic sulphureous thermal water Controls: no treatment	No significant variation in the average of the maximum percentage decreases in FEV1 compared to the baseline after inhalation of thermal water (−4.9 ± 1.5%) and after inhalation of physiological solution (−4.7 ± 0.8%); significant variation in the average of the maximum percentage decreases in FEV1 compared to the baseline one minute after inhaling thermal water (−4.0 ± 1.7%, *p* < 0.05); no significant change in the maximum percentage reduction in PEFR (peak expiratory flow rate) compared to the baseline value (−1.4 ± 1.8%)	High Bias Risk
Contoli et al. 2013 Italy Randomized double-blind clinical trial Sulphurous water of Riolo Terme	40; 20 interventions and 20 controls; 69.9 ± 1.0 years; 11 Females (27.5%) and 29 Males (72.5%)	Chronic obstructive pulmonary disease (COPD)	Evaluation of the effects of sulphurous thermal water inhalation on symptoms (CAT score), on the airway oxidative burst, on sputum total inflammatory cell counts and on lung function	Interventions: inhalation of sulphurous water in 2 different formulations: (1) 500mL warm inhalation, and after 20 min, (2) 5mL conventional aerosolisation Controls: inhalation of isotonic saline in 2 different formulations: (1) 500mL warm inhalation, and after 20 min, (2) 5mL conventional aerosolisation	Significant reduction in the CAT score in interventions (*p* < 0.05) and not in controls; inhibition of O₂ˉ production by the treatment, persisting for one month (*p* < 0.001); no difference in the total sputum cell counts in interventions and increase in controls (*p* < 0.05); no significant change in lung function parameters at the one-month follow up	Low Bias Risk
Guarnieri et al. 2010 Italy Randomized cross-over single-blind clinical trial Salt-bromide-iodine thermal water of Abano Terme-Montegrotto	13; 69.0 ± 3.0 years; 3 Females (23.1%) and 10 Males (76.9%)	Chronic obstructive pulmonary disease (COPD)	Evaluation of the effects of thermal water inhalation on chronic bronchitis symptoms and dyspnea, using the Communauté Européenne du charbon et de l’Acier (CECA) questionnaire, pulmonary function tests (FEV1) and EBC (exhaled breath condensate) collection (pH and leukotriene B4, LTB 4)	Interventions: inhalation of thermal water at a temperature of approximately 37° C and nebulized with an output of 50 ml/min, once a day for 20 min, for 2 weeks + after 4 weeks of wash-out period, inhalation of normal saline water at a temperature of approximately 37° C and nebulized with an output of 50 ml/min, once a day for 20 min, for 2 weeks Controls: inhalation of normal saline water, both administered at a temperature of approximately 37° C and nebulized with an output of 50 ml/min, once a day for 20 min, for 2 weeks + inhalation of thermal water at a temperature of approximately 37° C and nebulized with an output of 50 ml/min, once a day for 20 min, for 2 weeks	No significant differences detected in lung function and dyspnea score; no significant change of EBC LTB 4 concentration after both treatments; significant decrease of non-deaerated EBC pH in interventions (from 7.45, IQR 6.93–7.66, to 6.99, IQR 6.57–7.19; *p* = 0.05); no significant change in deaerated EBC pH in interventions (from 7.58, IQR 7.26–7.71, to 7.24, IQR 6.98–7.75); no significant effects on non-deaerated and deaerated EBC pH in controls (from 7.23, IQR 6.66–7.54 to 7.30, IQR 6.98–7.49, from 7.47, IQR 7.29–7.79, to 7.45, IQR 7.34–7.87, respectively)	Medium Bias Risk
Pellegrini et al. 2004 Italy Randomized single blind clinical trial Salt-bromide-iodine thermal water ( Abano-Montegrotto)	39; 20 interventions and 19 controls; 44–76 years; 4 Females (10.3%) and 35 Males (89.7%)	Chronic obstructive pulmonary disease (COPD)	Evaluation of the effects of crenotherapy with salt-bromide-iodine on quality of life (St. George’s respiratory questionnaire), lung function (spirometry) and airway inflammation	Interventions: inhalation of thermal water once daily for 20 min for 2-weeks Controls: inhalation of normal saline water once daily for 20 min for 2-weeks	Significant improvement of quality of life (St. George’s respiratory questionnaire) in interventions; no changes in pre- and post-salbutamol lung volumes in both groups; significant increase (*p* < 0.05) in total sputum cell concentration in both groups; small but significant decrease (*p* < 0.05) in percentages of sputum neutrophils (*p* < 0.01) and a parallel increase in macrophages (*p* < 0.01) in interventions	Medium Bias Risk
Fanfulla et al. 1997 Italy Randomized single-blind clinical trial Alkaline-earthy-sulfate water of San Pellegrino Spa Sponsorized	28; 64.6 ± 8.2 years	Chronic obstructive pulmonary disease (COPD)	Evaluation of the short-term effectiveness of thermal water treatment in COPD using physical activity tolerance (6-minute walk test), FEV-1, Tiffenau index (TI) (spirometry) and as parametres	Interventions: (1) usual therapy + full course of inhalation therapy with thermal water (inhalations, aerosol and positive pressure lung ventilation); (2) usual therapy + full course of inhalation therapy with thermal water + administration of bronchodilator drugs by intermittent positive pressure ventilation Controls: usual therapy	Improvement of walking distance values only in interventions (2); statistically significant improvements in FEV-1 in both interventions [interventions (1) 6 ± 5.1; interventions (2) 10.9 ± 7.9, *p* < 0.05] and no difference in controls; statistically significant improvements in TI in both interventions (*p* < 0.05) an no differences in controls	Medium Bias Risk
Corsico et al. 1994 Italy Randomized clinical trial Salso-bromo-iodine water of Euganean basin Sponsorized	28; 14 interventions and 14 controls; 59.5 ± 8.3 years; 18 Females (64.3%) and 10 Males (35.7%)	Chronic bronchitis	Evaluation of the effects of thermal therapy with salso-bromo-iodine water on sputum LDH values and LDH values in bronchial mucus	Interventions: inhalation of aerosol of salso-bromo-iodine water once a day for 12 days Controls: inhalation of physiological water once a day for 12 days	Decrease of sputum LDH values (from 2124 ± 798 to 1203 ± 814) after 12 days in intervention group and no statistically significant difference in controls; decrease of LDH values bronchial mucus in interventions (from 2410 ± 823 UI/L to 528 ± 380 UI/L) and no statistically significant difference in controls	Medium Bias Risk
Corradi et al. 2012 Italy Quasi-experimental trial Salt-bromide-iodine thermal water of Terme of Monticelli (Parma)	42; 70.8 ± 9.3 years; 16 Females (38.1%) and 26 Males (61.9%)	Alveolar (Silicosis, Asbestosis, Silicoasbestosis) or bronchial diseases (Chronic Bronchitis, Asthma)	Evaluation of the effects of salt-bromide-iodine thermal water inhalation on lung function parameters and on biomarkers detected in biological matrices, collected noninvasively (exhaled breath and exhaled breath condensate - EBC)	Interventions: 12 days inhalation treatment with salt-bromide-iodine water Controls: no treatment	Higher spirometric data after thermal treatment compared to baseline values, but only significant increase of mean PEFR value in bronchial diseases (y (4.8 ± 1.7 versus 5.1 ± 1.8; *p* < 0.01); significant increase of the index FEV1/FVC in patients with airflow obstruction compared to baseline value (0.6 ± 0.08 versus 0.65 ± 0.1; *p* < 0.05); no significant differences for: levels of FeNO at different flow rates (50, 100, 150, 350), for maximum total airway nitric oxide flux and alveolar nitric oxide concentration, for H₂O₂-EBC concentrations, for EBC Metals Concentrations (in subgroup of patients with significant smoking history) and for EBC anions concentrations in both groups of patients before and after thermal water inhalation	Medium Bias Risk

^*^ Miraglia Del Giudice et al. 2011 reported data on both upper and lower respiratory diseases and, thus, the article was included in both Tables 1 and 2

## Results

**Fig. 1:**
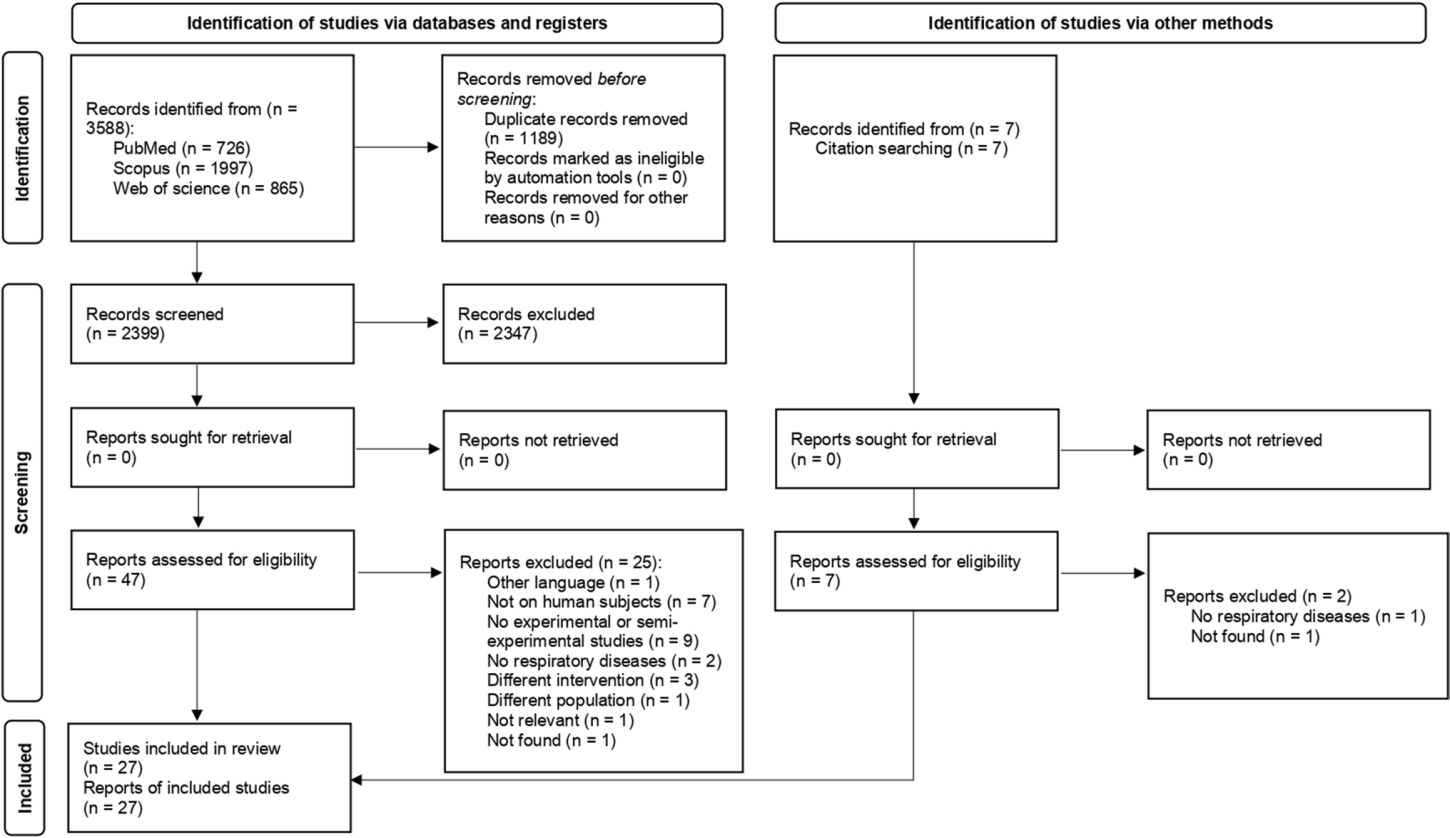
- Prisma flow chart describing the research strategy

The review process is described in Fig. 1. The search started using the search string on 3 different databases (PubMed, Scopus and Web of Science) obtaining 3588 records. After duplicates removal (1189), 2399 articles remained. Title and abstract analysis detected 52 records. Of these, 25 were excluded after full texts reading for not meeting inclusion criteria and reasons of exclusion reported and described in the Prisma 2020 flowchart (Fig. 1). At the end of the process, 27 articles were considered eligible and finally included in the systematic review.

The data extracted from the full-text reading and analysis of the included studies are summarized in Table 1 (upper respiratory diseases) and 2 (lower respiratory diseases). Attention was focused on the bibliographical data, the countries in which the studies were performed, possible conflict of interest (as undisclosed sponsorship), the study designs, the characteristics of the population studied (e.g. sex, age), the types of respiratory diseases, the characteristics of the TW and the hosting spafacilities the descriptions of treatments, the outcomes investigated, the results obtained and the risk of bias.

Of the 27 studies included in our review, 18 were clinical trials: seven of them are randomized but not blinded (Barbieri et al. 2002; Corsico et al. 1994; Cristalli et al. 1996; Miraglia Del Giudice et al. 2011; Passali et al. 2008a, b, 2016), six of them were double-blinded (Contoli et al. 2013; Ottaviano et al. 2011, 2012; Salami et al. 2010; Marullo and Abramo 1999, 2000), three single blinded (Fanfulla et al. 1997; Pellegrini et al. 2005; Varricchio et al. 2013) one of them was single-blind and also cross over (Guarnieri et al. 2010) and one of them was not randomized (Pollastrini et a. 1996). The remaining 9 articles were quasi-experimental studies (Ciprandi et al. 2016; Magrone et al. 2016; Passali et al. 2013; Passariello et al. 2012; Corradi et al. 2012; Pagani et al. 2011; Vassallo et al. 2009; Staffieri et al. 2007; Sanguinetti et al. 1990).

**Fig. 2:**
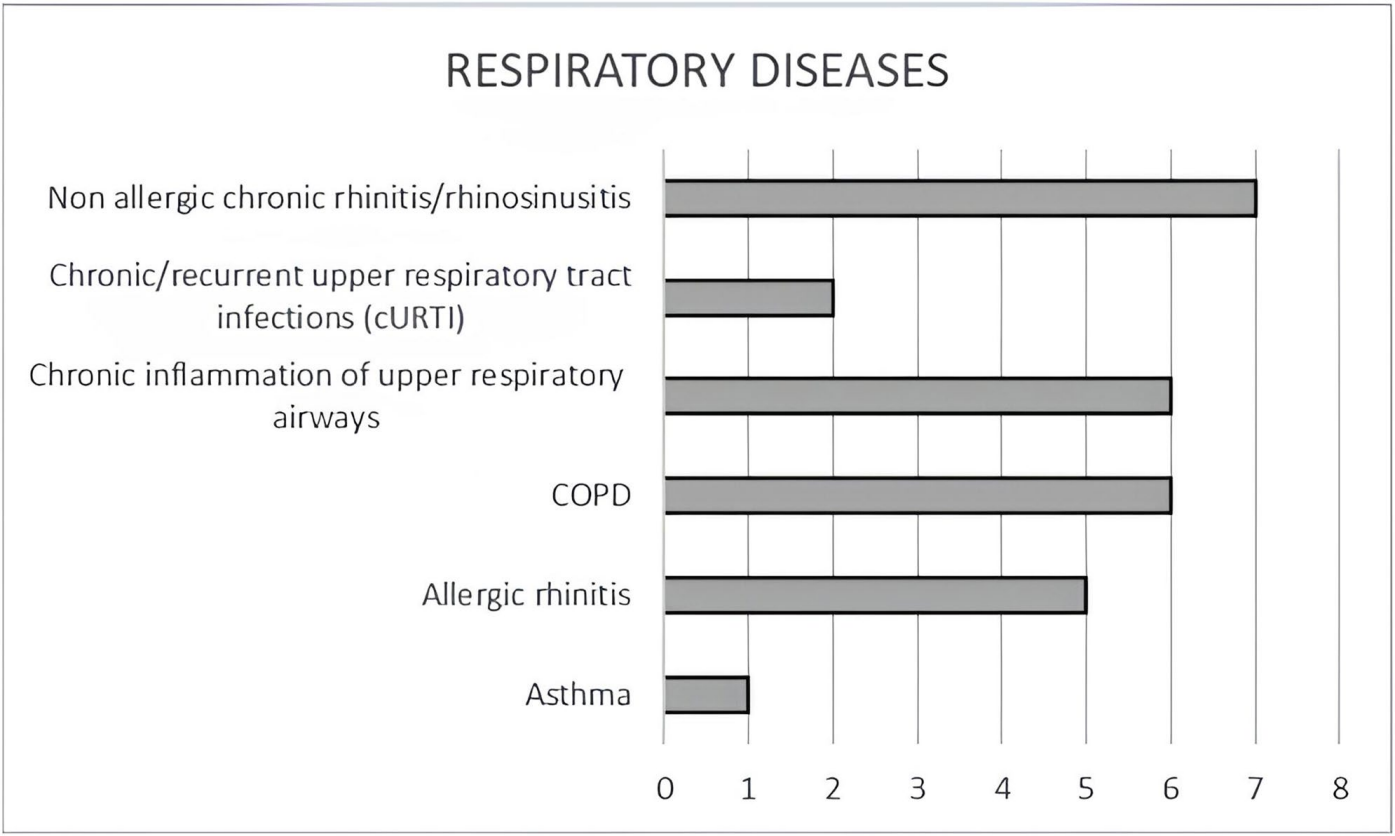
**- **Types of respiratory diseases studied in the included articles [Miraglia Del Giudice et al. (2011) was included both in the articles studying allergic rhinitis and asthma; Corradi et al. (2012), was included both in the articles studying COPD and asthma]

The main diseases examined were non-allergic chronic rhinitis, Chronic obstructive pulmonary disease (COPD) and allergic rhinitis as shown in Fig. 2.

All studies were carried out in Italy over a period ranging from 1990 to 2016. Most of the studies fosused on TW belonging to the Euganean basin. Inhalation was the most often used technique, while only three trials employed nasal irrigation (Ottaviano et al. 2011, 2012; Passali et al. 2013) and two used endonasal spray (Barbieri et al. 2002; Passali et al. 2008a).

In the treatment of chronic non-allergic rhinitis/rhinosinusitis, a significant improvement in symptoms such as headache (Passali et al. 2008a) and nasal obstruction (Passariello et al. 2012) is reported, as well as in parameters such as nasal resistance (Ottaviano et al. 2011, 2012; Staffieri et al. 2007), number of sleep arousal (Passali et al. 2008b), reduction of TNF-alpha, calprotectin and hBD-2 concentration (Passariello et al. 2012) and reduction of serum IgE concentration (Salami et al. 2010).

In the treatment of COPD, an improvement in general symptoms and certain parameters was noted, as in allergic diseases, but in one study where no changes in lung function parameters were noted (Contoli et al. 2013), in contrast to other two studies, in which a significant improvement in FEV-1 (Fanfulla et al. 1997) and of the index FEV1/FVC (Corradi et al. 2012) was reported in case group. In one article, a significant increase in macrophages in the sputum of treated subjects was reported (Pellegrini et al. 2005). An improvement in spirometric values is also found in the treatment of asthma in interventions (Sanguinetti et al. 1990). Another parameter investigated for asthma was FeNO, for which no significant change in interventions (Corradi et al. 2012) and a significant reduction in both interventions and controls (Miraglia Del Giudice et al. 2011) was demonstrated.

All studies agreed that using thermal water to treat allergy-related diseases (Pagani et al. 2011; Ciprandi et al. 2016; Barbieri et al. 2002; Miraglia del Giudice et al. 2011) improved symptoms like sneezing, itching, and nasal obstruction, with the exception of the results recovered by Passali et al. (2016). However, one study, reported an increase of IgA in the intervention group and a decrease in the control group (Barbieri et al. 2002). Besides, in another, there was no difference in FEV-1 between the two groups (Miraglia del Giudice et al. 2011). In addition to symptomatology, treatment with TW has been shown to produce a significant improvement in mucociliary function and nasal cytology (Passali et al. 2013).

In agreement with the other pathologies treated, in chronic inflammations of the upper respiratory tract, TW were also reported an improvement of signs and symptoms (Vassallo et al. 2009), a decrease in squamous metaplasia in interventions (Cristalli et al. 1996), an improvement of mucociliary transport (Pollastrini et al. 1996), nasal flow, nasal cytology and reduction of nasal resistance (Marullo and Abramo 1999, 2000; Staffieri and Abramo 2007).

With regard to chronic/recurrent upper airways infections, significant improvement was also reported mainly in terms of reduction of infectious events (Magrone et al. 2016; Varricchio et al. 2013).

According to the checklist CLEAR NTP for the risk of bias and quality of the studies, 16 of selected articles have a medium risk of bias, five have a high risk, and six have a low risk.

## Discussion

The present systematic review was conducted for evaluating the efficacy of TW to manage signs and symptoms of respiratory diseases. Indeed, the use of TW was evidenced as a potentially effective method in the treatment of respiratory diseases and, probably, the effect is related to the physical and chemical characteristics of the water. In the 27 included studies considered for the systematic review specific frequent chemical elements in the compositions of TW have been identified. In fact, 14 studies used TW containing sulfur in a sulfide or sulfated form, 12 studies used waters containing sodium, and 10 studies used waters containing iodine and bromine. Three considered studies containing radioactive oligomineral water, while one study used CO₂-enriched thermal water. Different types of TW act differently depending on their biochemical composition. For example, the use of sulfurous waters leads to a reduction in mucus production and viscosity (Costantino et al. 2006). Moreover, the same waters, act by decreasing the secretion of elastase by neutrophils, which would lead to a reduction in respiratory inflammatory processes (Braga et al. 2010). Indeed, in the inflammatory process, elastase activity cohorts tissue regeneration (Lengas et al. 1994), an action that, if uncontrolled, causes tissue damage so as to aggravate inflammation. Sulfur TW (STW) are also able to reduce oxidative DNA damage, resulting in curative effects on the respiratory tract (Braga et al. 2013). In addition, the deposition of thermal water droplets in target areas of the nasal mucosa has also been evaluated through a 3D model, allowing to hypothesise the effectiveness of thermal treatments in respiratory diseases (Buijs et al. 2019). The respiratory diseases considered in the analysed studies are very heterogeneous, concerning chronic and chronic-recurrent processes. Regarding COPD, the production and action of oxidant molecules is important in the disease process (Psarras et al. 2005). For example, the study conducted by Contoli et al. (2013) found an inhibition of O2- production with an important reduction of CAT score in interventions. Thus, it’s possible to hypothesise that a reduction in oxidative agents impedes the pathogenetic process specific of COPD. Besides, Passariello et al. (2012) reported a significant decrease in the concentrations of TNF alpha, a major mediator of inflammation, Calprotectin, one of the intracellular molecules most commonly found in macrophages and neutrophils, and hBD-2, a molecule widely found in the serum of patients with chronic inflammatory diseases, was demonstrated in patients with chronic rhinosinusitis, with a synchronous reduction in symptoms with an improvement in quality of life. These two evidences allow us to hypothesise that the reduction of important mediators of inflammation leads to a decrease of inflammation in the distribution areas of TW aerosols. In addition, according to Lowery et al. (2013), the increased incidence of cURTI is linked to an altered pulmonary immune system function, and in a reviewed study, TW inhalation was shown to upregulate the cytokines produced by Th1 and Th2 and to increase IL-21 and IL-17 in serum with a reduction in the number of infectious episodes (Magrone et al. 2016). Thus, allowing us to assume that regularization of cell activities and of some specific immune system mediators may act as protective mechanisms against cURTI. Overall, the results of the included studies reported an improvement in muco-ciliary transport, an improvement of lung function, and a reduction of pro-inflammatory cells in the sputum of patients with respiratory diseases.

Although the results emphasize the beneficial effects of TW on respiratory diseases, this systematic review has some limitations. First, the results cannot be considered definitive as the studies analysed are all predating 2016, demonstrating the absence of recent studies. Secondly, grey literature was not considered because we only aimed at peer reviewed studies. Besides, we included articles published in Italian or English language; thus, it is possible that we excluded some relevant studies published in other language. However, as reported by a recent systematic review, limiting the systematic reviews to English language publications seems to determine little impact on the effect estimates and conclusions of systematic reviews (Dobrescu et al. 2021). In addition, the quality of the studies does not completely meet the bias criteria, with only six studies presenting a low bias risk. Moreover, all the considered studies have been conducted in Italy, perhaps due to higher concentration of spa areas in Europe (Tamburello et al. 2022) and the legal status of TW therapy as a method of treatment in Italy. An additional limitation of our review is the absence of a meta-analysis due to respiratory diseases heterogeneity, assessment methods, and results. However, the highlighted results and the fact that in almost all studies TW have been used as individual treatment increase the concreteness of its curative properties.

## Conclusion

The current systematic review has considered studies that have overall highlighted the beneficial effects of TW towards the investigated respiratory diseases. However, the quality of the studies does not allow a definitive judgement due to the inaccurate methodologies, study designs and data elaboration. Thus, further studies are required with more appropriate methodologies and advanced statistical analysis.

## Data Availability

The data that support the findings of this study are available from the corresponding author upon reasonable request.
